# Effect of Cosmetic Ceramics on Fracture Toughness of All-Ceramic Restorations

**Published:** 2018-05

**Authors:** Sibel Cetik, Marion Vincent, Ramin Atash

**Affiliations:** 1 Professor, Laboratory of Physiology and Pharmaceutics, School of Medicine, Université Libre de Bruxelles, Brussels, Belgium; Department of Stomatology and Dentistry, Erasmus Hospital, Université Libre de Bruxelles, Brussels, Belgium; 2 Dentist, Department of Stomatology and Dentistry, Erasmus Hospital, Université Libre de Bruxelles, Brussels, Belgium; 3 Professor, Department of Stomatology and Dentistry, Erasmus Hospital, Université Libre de Bruxelles, Brussels, Belgium

**Keywords:** Zirconium Oxide, Ceramics, Mechanical Stress

## Abstract

**Objectives:**

The use of zirconia as a framework for prosthetic restorations is increasing due to its favorable mechanical properties. Zirconia also has remarkable aesthetic properties when used as a framework and covered with a layer of cosmetic ceramic. The aim of this study was to compare the fracture toughness of three types of aesthetic ceramics, namely VITA VM®9, ceraMotion® Zr, and IPS e.max® Ceram.

**Materials and Methods:**

Three groups of aesthetic ceramics (n=10) were subjected to three-point bending tests. The force leading to fracture was recorded for each sample to measure the impact of the ceramic type on the solidity of the framework. The type of fracture has not been studied in this work. One-way analysis of variance (ANOVA) was used to statistically analyze the results.

**Results:**

The statistical analysis showed significantly different fracture toughness values among the three groups. IPS e.max® showed the lowest fracture toughness (25.42 MPa) compared to VITA VM®9 and ceraMotion® Zr (respectively 40.39 MPa; P<0.001, and 48.78 MPa; P<0.005).

**Conclusions:**

Within the limitations of the present study, it can be concluded that aesthetic ceramics play an important role in the fracture toughness of all-ceramic restorations.

## INTRODUCTION

Due to the development of new fabrication techniques and technologies such as computer-aided design/computeraided manufacturing (CAD/CAM), the use of zirconia-based ceramic, as a restorative dental material, is strongly growing because of its superior mechanical properties [1]. Since restorative techniques are constantly improving, dental restorations should match the increasing demands for aesthetic and durable restorations [1]. Over the years, many inconveniences have been raised by ceramo-metallic restorations, most of them due to the opaque metallic under layer with average aesthetic results such as gradual gingival discoloration in the anterior buccal zone. In addition, ceramo-metallic restorations are prone to corrosion [1]. Since 1958, manufacturers have been developing ceramo-ceramic restorations as an alternative to metal-ceramics. Currently, biomaterial studies in the field of ceramo-ceramics have achieved significant results [2,3]. Although many different materials are now available for the ceramic infrastructure, clinical experience has shown that only two of these materials match the main criteria of mechanical resistance, aesthetics, and ease of processing, namely lithium disilicate and zirconia [2,4, 5].

On the other hand, to ensure dental prosthesis sustainability, the veneering ceramic should reach high levels of aesthetic potential and a great reliability [3]. As zirconia-based structures are usually combined with veneering ceramics in all-ceramic restorations, the purpose of this study was to explore the mechanical behavior of three commercial ceramic veneers in the shape of bilayered zirconia-veneer specimens under three-point bending tests. Comparative studies of the three selected ceramics were also carried out.

## MATERIALS AND METHODS

Three groups of A2 shade veneering ceramics were studied including group 1: IPS e.max® Ceram (Ivoclar Vivadent Inc., Schaan, Liechtenstein), group 2: VITA VM®9 (Vita Zahnfab-rik, Bad Sackingen, Germany), and group 3: ceraMotion® Zr (Dentaurum GmbH & Co, Ispringen, Deutschland). The samples were analyzed by three-point bending tests in order to measure the effect of the veneering ceramic type on the fracture toughness of the framework.

To achieve this aim, 30 strips of zirconia (Zirlux® ST1, Henry Schein Inc., Melville, NY, USA) were prepared according to the instructions of the manufacturer. Each sample was trimmed from a block of zirconia in a cutting machine (Secotom-50, Struers GmbH, Maas-sluis, Netherlands) by a disk (Diamond Cut-off Wheel EOD15). Each specimen consisted of a plate of 0.5±0.1 mm thickness with the lateral dimensions of 25±1 mm × 3±0.1 mm.

The sintering process for Zirlux® ST1 started with a heating phase reaching 1530°C in 2 hours, followed by a cooling phase during which the temperature was decreased to 800°C in 1 hour and 5 minutes. The 30 specimens were divided into three groups of 10 samples. The samples in each group were veneered with one of the three mentioned veneering ceramics (A2 shade).

The center of each zirconia bar was first covered with a thin layer of a veneering ceramic, and then, was slightly sintered in order to obtain an intermediate layer which promotes the bond strength between the zirconia and veneering ceramic. The sample was then sintered for 15 minutes.

A thicker layer of ceramic was then applied to the sample which was then placed on a vibrator to condense the ceramic. Again, the duration of the sintering process was 15 minutes. The process ended with the glazing of the whole sample. Thus, three groups of 10 specimens were obtained:

Group I (n=10): Zirlux ®SL1 bar + ceraMotion® Zr (Dentin A2) veneering ceramic
Group II (n=10): Zirlux ®SL1 bar + VITA VM®9 (Dentin A2) veneering ceramic
Group III (n=10): Zirlux ®SL1 bar + IPS e.max® (Dentin A2) veneering ceramic

Flexural testing of the zirconia-based core materials was carried out by the Schwickerath test and by following the procedures and recommendations of the International Organization for Standardization (ISO 9693-1:2012) [6].

Three-point bending tests were conducted under displacement control in a universal testing machine (MTS systems Co., Eden Prairie, MN, USA). The sample holder included two support rollers, separated by a 20-mm distance, and one loading roller. All the specimens were tested at a crosshead speed of 1.5±0.5 mm/minute until the breaking point was reached.

The loads at fracture point were analyzed by using one-way analysis of variance (ANOVA) with a significance level of 5%. Calculations and statistical analyses were performed by using SPSS version 23 software program (IBM Co., Chicago, IL, USA).

## RESULTS

A comparative analysis of the fracture toughness of the three veneering ceramics adhered to zirconia is presented in this section.

**Fig. 1:**
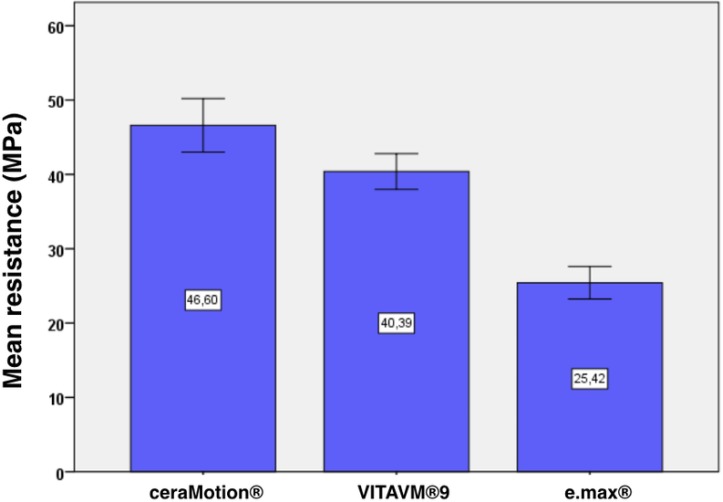
Representation of the mean fracture toughness (MPa) of ceraMotion® Zr, VITA VM®9, and IPS e.max® veneering ceramics (n=number of samples; the error bars represent 95% confidence interval (CI) of the mean)

The fracture toughness values of each group are presented in Figure 1 and Table 1.

**Table 1.** Mean fracture toughness (MPa) of ceraMotion® Zr, VITA VM®9, and IPS e.max® veneering ceramics

**Groups**	**Veneering ceramic**	**Number of samples**	**Fracture toughness (Mean±SD)**
1	ceraMotion® Zr	10	48.78 ± 10.42
2	VITA VM®9	10	40.39 ± 3.35
3	IPS e.max®	10	25.43 ± 3.05

SD=Standard Deviation

In the ceraMotion® Zr group, the mean fracture toughness was 48.78 MPa compared to 40.39 MPa in the VITA VM®9 group (mean difference=6.21 MPa, 95% confidence interval (CI)=1.74 to 10.67, P<0.005) and 25.43 MPa in the IPS e.Max® group (mean difference=21.17 MPa, 95% CI=16.71 to 25.64, P<0.001).

The mean fracture toughness in the VITA VM®9 group was 40.39 MPa compared to 25.43 MPa in the IPS e.max® group (mean difference=14.96 MPa, 95% CI=10.50 to 19.43, P<0.001).

Statistically significant differences were found between the ceraMotion® Zr group and the VITA VM®9 group (P<0.005), between the ceraMotion® Zr group and the IPS e.max® group (P<0.001), and between the VITA VM®9 group and the IPS e.max® group (P<0.001).

## DISCUSSION

Many parameters can influence the zirconia core-veneer ceramic interface such as the core roughness and surface energy, the presence of defects (bubbles) at the interface, wettability and viscosity of the veneering ceramic, the stress induced during the cooling phase due to the TEC (thermal expansion coefficient) mismatch between the veneer and core, and the flexural strength of the ceramic [3].

Three veneering ceramics adhered to zirconia were tested in the present study: a low-fusing glass ceramic (ceraMotion® Zr), a feldspathic high-fusing ceramic (VITA VM®9), and a low-fusing nanofluorapatite glass-ceramic (IPS e.max®) with high-fusing liners. The properties of these ceramics are summarized in Table 2.

**Table 2.** Specifications of ceraMotion® Zr, VITA VM®9, and IPS e.max® veneering ceramics

**Veneering ceramic**	**Type of material**	**TEC (×10−6 °C-1) (25–500°C)**	**Tg (°C)**	**Flexural Strength (MPa)**
ceraMotion® Zr	Glass	9.2	530	115
VITA VM®9	Feldspathic ceramic	9.0–9.2	600	100
IPS e.max®	Fluorapatite glass-ceramic	9.5 9.8 (liner)	490 645 (liner)	90 90 (liner)

TEC=Thermal Expansion Coefficient, Tg=Transition temperature

To eliminate the influence of surface roughness and surface energy of the framework, the same zirconia was used with each of the veneering ceramics: the zirconia bars were directly layered without sandblasting or grinding after sintering. No long-term cooling was performed in this study. The mechanism of ceramic-ceramic adhesion is not as clear as that of the metal-ceramic adhesion. When veneering ceramics are applied to alloys, the mechanical retention and chemical bond between the ceramic and oxide layer play a predominant role in adhesion [7,8]. The bonding mechanism between the veneer ceramic and zirconia is not completely understood. The high level of flexural strength measured in this study with regard to the non-sandblasted bars, from 43.9 MPa to 46.6 MPa for ceraMotion® Zr on non-colored zirconia, can orientate the bonding nature to chemical rather than to mechanical. Aboushelib et al [1] have measured a diffusion depth of 8 to 10 μm of porcelain components into the zirconia framework, demonstrating a chemical bonding between the veneering ceramic and zirconia. A study by Fischer et al [9] showed that mechanical surface treatments such as sandblasting do not improve the adhesion between the veneering ceramic and zirconia.

Within the cooling phase, the TEC mismatch between the veneering ceramic and zirconia core leads to the development of residual stresses. The concentration of this stress at the interface can lead to debonding [10]. Due to the viscoelastic properties of the veneering ceramic, a gradient of stress appears in the ceramic at glass transition temperature (Tg), and the ceramic becomes solid [10]. A TEC slightly lower than that of the framework is recommended for the veneering ceramic, which leads to a positive mismatch inducing a compressive stress in the veneering ceramic [10,11]. Stress distribution in a bilayered structure is not uniform; the compressive stress distributed in the ceramic layer is higher at the core-veneer interface and decreases toward the veneering surface leading to a slight tensile stress [10]. A tensile stress is not recommended because it reduces the strength of the veneering ceramic and can induce cracks [10].

The compressive stress generated in the veneering ceramic can strengthen the bilayered structure [4,5]. The degree of the compressive stress in the veneering layer influences the level of flexural strength [11]. Fischer et al [11] demonstrated that the TEC and Tg can influence the adhesion between the bilayered ceramic and zirconia. In this study, the TEC variations of the veneering ceramics between 25°C and 500°C were not significantly different for VITA VM®9 and ceraMotion® Zr ceramics; the values ranged from 9.0×10−6°C-1 to 9.2×10−6°C-1. For IPS e.max®, the TEC was slightly higher (9.5×10−6°C-1). The Tg ranged from 490°C (IPS e.max®) to 600°C (VITA VM®9) (Table 2). A complex residual stress can be generated in bilayered restorations by the firing processes of the veneering porcelain. Since a slow cooling can negatively affect the flexural strength of bilayered zirconia core and veneering ceramic [12], rapid cooling was performed in this study for all the veneering ceramics. Stress is generated by TEC mismatch, Tg level, and viscoelastic behavior of the ceramic influencing the level of stress [13]. The IPS e.max® with the highest TEC and the lowest Tg presents the lowest adhesion values when applied to non-colored zirconia. Despite the difference in the TEC and Tg, other parameters such as viscoelasticity, wettability, and volume shrinkage of the veneering ceramic also have to be taken into consideration [13]. CeraMotion® Zr, which exhibits the highest bonding strength when applied to zirconia, is a glass with a low firing temperature, whereas VITA VM®9, which is a feldspathic ceramic with a high firing temperature, presents a significantly lower adhesion value [13].

The wettability and viscoelastic structural relaxation of the ceramic veneer should be taken into account to explain the results of this study. To impede the effects of these variables, the use of a liner for increasing the flexural strength is not recommended for all types of ceramics. For example, IPS e.max® is used with a liner; however, this is not the case for ceraMotion® Zr ceramic.

Zirconia-based ceramics are the most promising materials for dental application with good mechanical properties and an excellent biocompatibility [14,15], and many research activities have been carried out on their interaction with different ceramic veneers [14,16, 17]. As zirconia-based structures are usually combined with veneering ceramics for the production of all-ceramic restorations, the mechanical behaviors of three commercial ceramic veneers were analyzed in this study by three-point bending tests of bilayered zirconia-veneer specimens, and comparative studies of the three selected ceramics were also carried out.

Although some authors have shown that the coloring process, by using a commercial coloring liquid, has no significant effect on the mechanical strength of zirconia specimens [14], all the veneering ceramics in the three groups of our study were A2 shade veneering ceramics.

In our study, significant differences in fracture toughness were observed among the three types of ceramics; however, some authors have not observed such differences [18].

IPS e.max® ceramic showed the lowest mean fracture toughness (25.43±3.05 MPa), whereas ceraMotion® Zr ceramic showed the highest fracture toughness (48.78±10.42 MPa). VITA VM®9 ceramic presented an average fracture toughness of 40.39±3.35 MPa.

Fischer et al [19] showed different values from ours when they compared ceraMotion® Zr and IPS e.max® ceramics. This difference may be attributed to the protocol they used which included the application of a liner on IPS e.max® ceramic [19].

A search of the literature shows that the mechanism of bonding between the zirconia and veneering ceramics has remained unknown, and the bond strength between the zirconia and porcelain is still lower than that between metal and porcelain [20].

Our results show that the type of the veneering ceramic plays a significant role in the failure mechanisms of all-ceramic restorations. The adhesion between ceramic and zirconia framework is still an issue influencing the long-term success of prosthetic restorations.

The best combinations of core and veneering ceramics should be further studied. Also, clinical studies with larger sample sizes and longer follow-up periods are required to investigate the possible influencing factors that may lead to technical failures.

## CONCLUSION

Our results indicate that more attention should be paid to the choice of veneering ceramic for dental crowns and bridges since significant differences have been found in the fracture toughness of different veneering ceramics.

## References

[1-] Aboushelib MN Kleverlaan CJ Feilzer AJ Microtensile bond strength of different components of core veneered all-ceramic restorations. Part II: Zirconia veneering ceramics. Dent Mater. 2006 9;22(9):857–63. 16376981 10.1016/j.dental.2005.11.014

[2-] Aboushelib MN Kleverlaan CJ Feilzer AJ Effect of zirconia type on its bond strength with different veneer ceramics. J Prosthodont. 2008 7;17(5):401–8. 18355163 10.1111/j.1532-849X.2008.00306.x

[3-] Benetti P Della Bona A Kelly JR Evaluation of thermal compatibility between core and veneer dental ceramics using shear bond strength test and contact angle measurement. Dent Mater. 2010 8;26(8):743–50. 20472281 10.1016/j.dental.2010.03.019

[4-] Taskonak B Mecholsky JJ Jr Anusavice KJ Residual stresses in bilayer dental ceramics. Biomaterials. 2005 6;26(16):3235–41. 15603818 10.1016/j.biomaterials.2004.08.025

[5-] Coffey JP Anusavice KJ DeHoff PH Lee RB Hojjatie B Influence of contraction mismatch and cooling rate on flexural failure of PFM systems. J Dent Res. 1988 1;67(1):61–5. 11039047 10.1177/00220345880670011201

[6-] ISO 9693-1:2012 Dentistry -- Compatibility testing -- Part 1: Metal-ceramic systems. Available at: https://www.iso.org/standard/54946.html/ Accessed December 19, 2017.

[7-] Mackert JR Jr Ringle RD Parry EE Evans AL Fairhurst CW The relationship between oxide adherence and porcelain-metal bonding. J Dent Res. 1988 2;67(2):474–8. 11039060 10.1177/00220345880670020801

[8-] Schweitzer DM Goldstein GR Ricci JL Silva NR Hittelman EL Comparison of bond strength of a pressed ceramic fused to metal versus feldspathic porcelain fused to metal. J Prosthodont. 2005 12;14(4):239–47. 16359480 10.1111/j.1532-849X.2005.00052.x

[9-] Fischer J Grohmann P Stawarczyk B Effect of zirconia surface treatments on the shear strength of zirconia/veneering ceramic composites. Dent Mater J. 2008 5;27(3):448–54. 18717175 10.4012/dmj.27.448

[10-] Swain MV Unstable cracking (chipping) of veneering porcelain on all-ceramic dental crowns and fixed partial dentures. Acta Biomater. 2009 6;5(5):1668–77. 19201268 10.1016/j.actbio.2008.12.016

[11-] Fischer J Stawarzcyk B Trottmann A Hammerle CH Impact of thermal misfit on shear strength of veneering ceramic/zirconia composites. Dent Mater. 2009 4;25(4):419–23. 18990436 10.1016/j.dental.2008.09.003

[12-] Göstemeyer G Jendras M Dittmer MP Bach FW Stiesch M Kohorst P Influence of cooling rate on zirconia/veneer interfacial adhesion. Acta Biomater. 2010 12;6(12):4532–8. 20601242 10.1016/j.actbio.2010.06.026

[13-] Chantranikul N Salimee P Biaxial flexural strength of bilayered zirconia using various veneering ceramics. J Adv Prosthodont. 2015 10;7(5):358–67. 26576251 10.4047/jap.2015.7.5.358 PMC4644776

[14-] Marrelli M Maletta C Inchingolo F Alfano M Tatullo M Three-Point Bending Tests of Zirconia Core/Veneer Ceramics for Dental Restorations. Int J Dent. 2013;2013:831976. 23533415 10.1155/2013/831976 PMC3596922

[15-] Piconi C Maccauro G Zirconia as a ceramic biomaterial. Biomaterials. 1999 1;20(1):1–25. 9916767 10.1016/s0142-9612(98)00010-6

[16-] Guazzato M Proos K Quach L Swain MV Strength, reliability and mode of fracture of bilayered porcelain/zirconia (Y-TZP) dental ceramics. Biomaterials. 2004 9;25(20):5045–52. 15109867 10.1016/j.biomaterials.2004.02.036

[17-] Rahbar N Yang Y Soboyejo W Mixed mode fracture of dental interfaces. Mater Sci Eng A. 2008 8;488(1–2):381–8.

[18-] Diniz AC Nascimento RM Souza JC Henriques BB Carreiro AF Fracture and shear bond strength analyses of different dental veneering ceramics to zirconia. Mater Sci Eng C Mater Biol Appl. 2014 5;38:79–84. 24656355 10.1016/j.msec.2014.01.032

[19-] Fischer J Stawarczyk B Sailer I Hämmerle CH Shear bond strength between veneering ceramics and ceria-stabilized zirconia/alumina. J Prosthet Dent. 2010 5;103(5):267–74. 20416409 10.1016/S0022-3913(10)60056-X

[20-] Miyazaki T Nakamura T Matsumura H Ban S Kobayashi T Current status of zirconia restoration. J Prosthodont Res. 2013 10;57(4):236–61. 24140561 10.1016/j.jpor.2013.09.001

